# Limited importance of the dominant-negative effect of *TP53 *missense mutations

**DOI:** 10.1186/1471-2407-11-243

**Published:** 2011-06-13

**Authors:** Ewelina Stoczynska-Fidelus, Malgorzata Szybka, Sylwester Piaskowski, Michal Bienkowski, Krystyna Hulas-Bigoszewska, Mateusz Banaszczyk, Izabela Zawlik, Dorota Jesionek-Kupnicka, Radzislaw Kordek, Pawel P Liberski, Piotr Rieske

**Affiliations:** 1 Department of Molecular Pathology and Neuropathology, Chair of Oncology, Medical University of Lodz, Czechoslowacka 8/10, 92-216 Lodz, Poland; 2 Department of Pathology, Chair of Oncology, Medical University of Lodz, Paderewskiego 4, 93-509 Lodz, Poland

**Keywords:** TP53, heterozygous mutation, dominant-negative effect, cancer cell lines

## Abstract

**Background:**

Heterozygosity of *TP53 *missense mutations is related to the phenomenon of the dominant-negative effect (DNE). To estimate the importance of the DNE of *TP53 *mutations, we analysed the percentage of cancer cases showing a single heterozygous mutation of *TP53 *and searched for a cell line with a single heterozygous mutation of this gene. This approach was based on the knowledge that genes with evident DNE, such as *EGFR *and *IDH1*, represent nearly 100% of single heterozygous mutations in tumour specimens and cell lines.

**Methods:**

Genetic analyses (LOH and sequencing) performed for early and late passages of several cell lines originally described as showing single heterozygous *TP53 *mutations (H-318, G-16, PF-382, MOLT-13, ST-486 and LS-123). Statistical analysis of IARC *TP53 *and SANGER databases. Genetic analyses of N-RAS, FBXW7, PTEN and STR markers to test cross-contamination and cell line identity. Cell cloning, fluorescence-activated cell sorting and SSCP performed for the PF-382 cell line.

**Results:**

A database study revealed *TP53 *single heterozygous mutations in 35% of *in vivo *(surgical and biopsy) samples and only 10% of cultured cells (*in vitro*), although those numbers appeared to be overestimated. We deem that published *in vivo TP53 *mutation analyses are not as rigorous as studies *in vitro*, and we did not find any cell line showing a stable, single heterozygous mutation. G16, PF-382 and MOLT-13 cells harboured single heterozygous mutations temporarily. ST-486, H-318 and LS-123 cell lines were misclassified. Specific mutations, such as R175H, R273H, R273L or R273P, which are reported in the literature to exert a DNE, showed the lowest percentage of single heterozygous mutations *in vitro *(about 5%).

**Conclusion:**

We suggest that the currently reported percentage of *TP53 *single heterozygous mutations in tumour samples and cancer cell lines is overestimated. Thus, the magnitude of the DNE of *TP53 *mutations is questionable. This scepticism is supported by database investigations showing that retention of the wild-type allele occurs with the same frequency as either nonsense or missense *TP53 *mutations.

## Background

Single heterozygous mutations of *TP53 *are associated with gain-of-function (GOF) phenomena and, especially, the dominant-negative effect (DNE) [1,2]. Nonetheless, GOF can be attributed to hemizygous or homozygous, as well as heterozygous, mutations [3]. DNE may be linked to many processes, including the effectiveness of chemotherapy and the failure of *TP53 *gene therapy [4,5]. Dominant-negative *TP53 *mutations are frequently described as very strong, completely abolishing the function of the wild-type protein [6]. Nevertheless, certain observations undermine the importance of the dominant-negative effect of missense heterozygous mutations. According to Dearth *et al.*, approximately 60% of tumours with a missense mutation in *TP53 *have lost the wild-type allele, making a case against the absolute importance of the dominant-negative effect [7]. Moreover, Heyne *et al. *demonstrated the resistance of mitochondrial *TP53 *to dominant inhibition [8]. Li-Fraumeni patients' fibroblasts, exhibiting single heterozygous mutations, did not demonstrate clear alterations in vital functions [9]. The majority of work focusing on the dominant-negative effect and gain-of-function were performed under artificial model conditions using plasmids to express wild-type and mutated *TP53*. [10,11]. Other opportunities were offered by a rodent animal model [12]; however, the mouse model does not represent an exact human counterpart. To this end, we proposed an alternative approach to evaluate the significance of *TP53 *mutations DNE. Cell lines directly derived from human cancers with heterozygous *TP53 *mutations may be more relevant, and thus, we searched for cell lines with a single heterozygous *TP53 *mutation. Because haplo-insufficiency is not attributed to *TP53*, the percentage of cases demonstrating single heterozygous mutations can be used to estimate the importance of *TP53 *mutations DNE. Obviously, genes such as *IDH1 *and *EGFR*, for which a DNE is clearly observed, exhibit almost exclusively single heterozygous mutations [13,14]. Moreover, studies report differences and gradations for specific mutations [7,15]. This prompted us to perform a more complex analysis of databases and cell lines to estimate the percentage of single heterozygous *TP53 *mutations and, thus, the importance of *TP53 *mutations DNE. Our approach is in accordance with the suggestion of Olivier's group to consider the percentage of cases showing loss of heterozygosity (LOH) in the estimation of DNE importance [15]. As they have hypothesized, thorough assessment of the role of DNE in cancer development requires knowledge of the status of 17p LOH in tumours [15]. Furthermore, apart from LOH status, we have also analysed the occurrence of double mutations of *TP53*. Moreover, we have broadened the spectrum of results obtained from databases by analysing several cell lines *in vitro*.

## Methods

### Cultured Samples

The study included human cancer cell lines, cultured cells and corresponding tumour samples. Commercially available human cancer cell lines PF-382 and MOLT-13 were obtained from German Collection of Microorganisms and Cell Cultures (DSMZ), Braunschweig, Germany. Cell lines ST-486 and LS-123 were obtained from American Type Culture Collection (ATCC) Manassas, USA. H-318 mouse cell line was kindly provided by Professor Guillermina Lozano, Department of Cancer Genetics, The University of Texas M. D. Anderson Cancer Center, Houston. Cells were cultured in RPMI 1640 or MEM supplemented with 10% FBS (PAA, Linz, Austria) and penicillin/streptomycin/glutamin (GIBCO BRL, Paisley, UK), in 5% CO_2_. G-16 cultured cells were established from a surgically resected tumour from a patient diagnosed with glioblastoma. Archival paraffin sections of the tumour, from which G-16 cells were derived, were obtained from the Department of Pathology, Medical University of Lodz. G-16 cells were cultured in MEM supplemented with 10% FBS (PAA, Linz, Austria) and penicillin/streptomycin/glutamin (GIBCO BRL, Paisley, UK), in 5% CO_2_.

### Database Analysis

Our analysis of *TP53 *mutation status in human cancer cell lines and tumour samples was performed using two databases: the Sanger Institute Catalog Of Somatic Mutations In Cancer (COSMIC) that gathers information on genetic alterations (taken from the literature and in-house sequencing in human tumour samples and tumour cell lines), and the IARC *TP53 *Mutation Database that compiles all *TP53 *gene variations identified in human populations and tumour samples [15-18]. Analysis was confirmed using the *TP53 *Mutation Handbook - the last release of the UMD_p53 database, that collects information on the *TP*53 status in cell lines published between 1989 and 2008 [19]. The data were organized into 2 × 2 tables according to given variables and Χ^2 ^(chi squared) test was performed.

### DNA and RNA Extraction

Total cellular DNA and RNA were isolated from cell lines, cultured samples, frozen tissues (stored at -80°C) and frozen leukocytes of peripheral blood obtained from healthy volunteers and the patients using AllPrep DNA/RNA Mini Kit (Qiagen, Germany) according to the manufacturer's protocol. RNA samples were treated with DNase. RNA and DNA concentrations were measured spectrophotometrically. 100 ng of total RNA was reversetranscribed into single-stranded cDNA in a final volume of 40 μl containing 50 mM DTT, 1.5 μg oligo(dT), 0.5 mM dNTP, 40 units RNase inhibitor and 200 units M-MLV reverse transcriptase (Promega).

### DNA Extraction from Archival Formalin-fixed Paraffin-embedded Blocks

Specimens from a patient diagnosed with glioblastoma (classified according to the World Health Organization criteria for the classification of brain tumours) were obtained from the Department of Pathology, Medical University of Lodz. Tissue samples were fixed with 4% neutralized formalin and embedded in paraffin. Before tissue processing, histopathological examination of those specimens with H&E staining was necessary to confirm the target areas of tissue for DNA extraction. The same areas in the paraffin blocks were matched to accurately locate the target tissue for scraping. Two different marked target areas from paraffin block were carefully scraped with the surgical blade, to a maximum depth of 1 mm, to avoid contamination with underlying normal tissue. The scraped tissues were collected into 2 ml Eppendorf tubes, de-paraffinized with xylene, washed with ethanol and dried. 200 μl lysis buffer containing approximately 1 μg/μl KCev PRoteinase (Roche Diagnostics, Mannheim, Germany) was added to each tube and digested at 55°C overnight. DNA was then purified and extracted with phenol, phenol/chloroform mixture and chloroform, precipitated with 98% ethanol and dried in a speed vacuum. The obtained DNA was dissolved in 20 μl TE buffer.

### Loss of Heterozygosity Analysis (STR Analysis)

Loss of heterozygosity analysis (LOH) was performed using paired tumour specimens and corresponding peripheral blood samples. The following LOH markers were used: D17S1828, D17S976. Twenty additional markers were used for cross contamination analysis. The forward primers were 5'-end-fluorescence-labeled. PCR was performed in thermocycling conditions individually established for each pair of primers. Approximately 0.5 μl of each PCR product was denatured and gel electrophoresis in LiCor automatic sequencer system (Lincoln, NE) was applied for the separation and analysis of PCR-generated alleles. LOH was assigned when the intensity of the tumour alleles differed by at least 50% from that observed in the corresponding control DNA. STR analysis allowed to exclude possible cross contamination of analysed cell lines excluding H-318 (murine cell line).

### Single Strand Conformation Polymorphism (SSCP)

Exon 8 of the *TP53 *gene was amplified by PCR on DNA template in standard conditions 32x (92°C, 55°C, 72°C) using the 5'-end-fluorescence-labeled primers and mutations were detected by SSCP analysis. For SSCP analysis, PCR product was mixed with gel loading buffer (95% formamide, 20mM EDTA, 0.05% bromophenol blue and 0.05% xylene cyanole). The mixture was heat-denatured at 100°C for 5 min and rapidly chilled on ice. Denatured products were loaded on to the polyacrylamide gel supplemented with 10% glycerol and gel electrophoresis in LiCor automatic sequencer system (Lincoln, NE) was applied for the analysis.

### *TP53 *DNA and cDNA Sequencing

Exons from 2 to 11, introns from 4 to 8 and 3'UTR region of the *TP53 *gene were amplified by PCR on DNA template in standard conditions 32x (92°C, 55°C, 72°C) and sequenced using the dideoxy termination method and SequiTherm Excel DNA Sequencing Kit (Epicentre Technologies) following the protocol of manufacturer. Exons 5-8 of the *TP53 *gene were amplified by PCR on cDNA template as described before and sequenced using the dideoxy termination method and SequiTherm Excel DNA Sequencing Kit (Epicentre Technologies) [20]. To verify the results of sequencing the semiquantitative densitometric analysis was performed. The intensity of wild-type and mutated bands was estimated by comparing them to the neighbouring bands in the same sequencing lane used as a reference. Primer sequences provided as supporting information.

### Single Cell-cloning

In order to yield a homogeneous cell population, several cell lines were established by single-cell cloning from the primary culture of PF-382 cells provided by German Collection of Microorganisms and Cell Cultures, Braunschweig, Germany. Cloning was performed by limiting dilution into 96-well plates. Clonality was assessed immediately by microscopic observation to identify wells containing only a single cell. Among the clones, two rapidly growing lines, cell line A and B, were selected for further analysis based on DNA and cDNA sequencing, and compared with primary PF-382 cells and each other.

### Cell Sorting

PF-382 cell line was sorted under non-sterile conditions to retrieve subpopulations for DNA extraction and for morphological analysis. Cells from culture were centrifuged for 5 min at 1 500 rpm, washed three times in PBS at 37°C for 5 minutes. After this, cells were fixed with 4% paraformaldehyde at 4°C for 15 minutes and again centrifuged for 5 min at 1 500 rpm and then washed three times in PBS at 4°C for 5 minutes. For sorting, fixed cells were resuspended in 3 ml PBS. Light scatter parameters were used to establish sorting gates to distinguish the subpopulations of viable, apoptotic and dead cells. The analysis and separation of 1 000 000 cells was performed using FACSAria II cell sorter (BD Biosciences).

## Results

### Database Analysis

**Figure 1:**
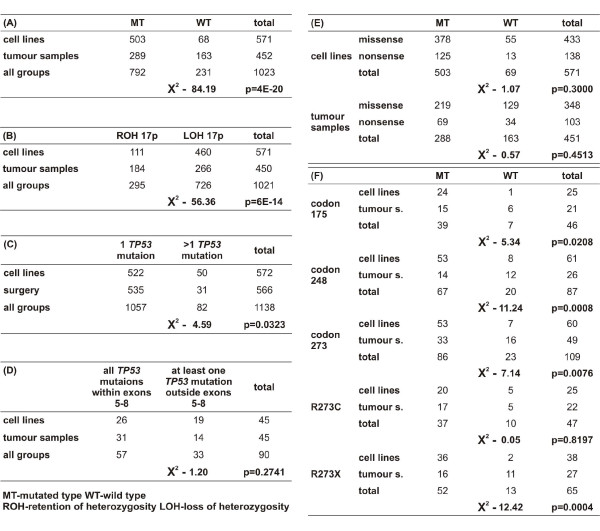
**The statistical analyses of databases**. **(A) **A comparison of *TP53 *wild type allele retention occurrence in human cancer cell lines with samples from surgeries and biopsies (marked as tumour samples) showing mutation, Χ^2 ^test was performed for cell lines *vs *tumour samples. **(B) **A comparison of ROH/LOH 17p occurrence in human cancer cell line showing mutation and tumour samples, Χ^2 ^test was performed. **(C) **A comparison of incidence of 1/more than 1 *TP53 *mutation in human cancer cell lines and tumour samples, Χ^2 ^test was performed. **(D) **A comparison of *TP53 *wild type allele retention occurrence in human cancer cell lines and tumour samples with the distinction between missense and nonsense mutation (sample was categorized as "missense" in case of at least 1 missense mutation, while "nonsense" with no missense mutations), Χ^2 ^test was performed for cell-lines and tumour samples separately. **(E) **A comparison of incidence of at least one *TP53 *mutation outside exons 5-8 versus all *TP53 *mutations within exons 5-8 in human cancer cell lines and tumour samples showing 2 or more *TP53 *mutations, Χ^2 ^test was performed. **(F) **A comparison of *TP53 *wild type allele retention occurrence in human cancer cell lines and tumour samples with mutations characteristic for dominant-negative effect (codons 175, 248, 273, with distinction for R273C, as with no DNE, and other types of mutation of codon 273 - marked R273X), Χ^2 ^test was performed for each analysis.

Analysis of the Sanger and IARC databases with respect to *TP53 *status in surgical and biopsy samples and in cultured cells yielded the results presented in Figure 1. Most importantly, the percentage of surgical and biopsy specimens described as single heterozygous was very high (35%) compared to cultured cancer cell lines supposedly carrying such a mutation (10%) (Figure 1A, 1B). Cell lines show a lower proportion of wild-type *TP53 *retention because of more frequent 17p LOH and second heterozygous *TP53 *mutations (Figure 1C). The percentage of samples with at least one mutation outside exons 5-8 is higher in cancer cell lines, with at least two mutations, than in tumour samples with two mutations (42% in cell lines and 31% in tumour samples - not statistically significant) (Figure 1D). The frequency of retention of the wild-type allele in cancer cell lines with a missense mutation is almost the same as the frequency amongst cancer cell lines with a nonsense mutation (Figure 1E). Additionally, our database analysis performed two years ago demonstrated that several cell lines harbour a single heterozygous mutation. Currently, the following cell lines are described as showing two, three or more mutations: CMK, Cha-Go-K-1 and NCI-H661, respectively. The status of the MOLT-16, KM-12, SK-LMS-1, J-82, Daudi and Raji cell lines remains controversial; the Sanger database lists these cell lines as possessing a single mutation, whereas the *TP53 *Mutation Handbook shows them as having two or more mutations.

Additionally, database analysis provided important information regarding *TP53 *hot-spot mutations (Figure 1F). Codon 248 exhibited the highest percentage of *in vivo *single heterozygous mutations, whereas the percentage of *in vitro *mutations in this codon was as low as any mutation of *TP53*. Codon 175 demonstrated a very low percentage of single heterozygous mutations *in vitro *and an average percentage *in vivo*. Analysis of codon 273 provided additional information, specifically, that mutation R273C was a single heterozygous mutation in approximately 20% of cell lines, both *in vivo *and *in vitro*. For other mutations of this codon, the incidence was 42% and 5% *in vivo *and *in vitro*, respectively.

### G-16 Analysis

**Figure 2:**
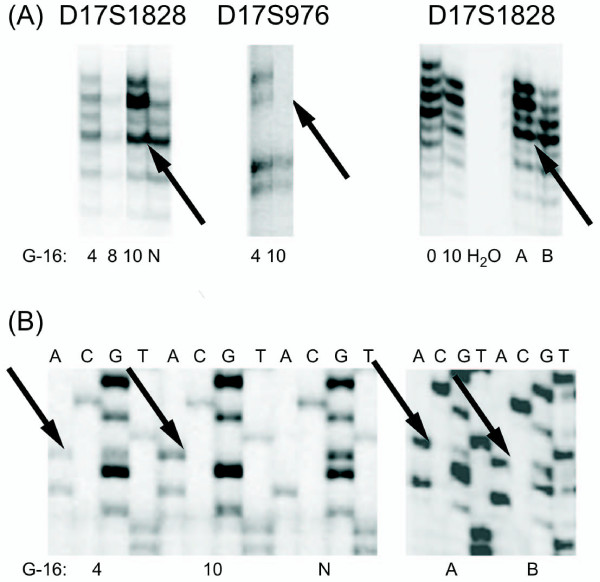
**Molecular analysis of G-16 glioblastoma cultured cells and paraffin block fragments**. **(A) **Examples of LOH analysis showing deletion in 17p region at passage 10 (D17S1828 and D17S976 markers were used) and occurrence of the subpopulation of cells with 17p LOH *in vivo *(D17S1828 marker was used). Only a trace of the lost allele is observed in the 10 passage of G-16 cultured cells and in the paraffin block fragment A. The lost allele is marked with an arrow. **(B) ***TP53 *DNA sequencing results. The mutated nucleotide (*TP53 *exon 8, codon 272, GTG > ATG, Val > Met) is marked with arrows. 1) G-16 cultured cells at passage 4, G and A nucleotides are both detected, representing a heterozygous mutation. 2) G-16 cultured cells at passage 10, no wild type, only mutated nucleotide is detected. 3) Normal control template, only wild type DNA is detected. 4) G-16 paraffin block fragment A, both nucleotides are detected but G nucleotide is faint, suggesting a hemizygous mutation. 5) G-16 paraffin block fragment B, G and A nucleotides are both detected, representing a heterozygous mutation. N - corresponding normal tissue (blood) or normal, control template; A - G-16 paraffin block fragment A; B - G-16 paraffin block fragment B; 0, 4, 8, 10 - numbers of passages.

A surgical specimen of glioblastoma (GBM) was analysed by sequencing and LOH analysis. A mutation in codon 272 was observed with both wild-type and mutated templates, although the band representing the latter was faint. In addition, retention of heterozygosity of 17p was shown by means of D17S1828 and D17S976. Cells grown under standard conditions showed the 272 mutation, but no 17p LOH at the 0 passage. However, after 4 passages, the mutated template became more apparent, and 17p LOH became detectable. After 10 passages, only the mutated template was present and LOH analysis demonstrated only one allele (Figure 2). DNA was isolated from a paraffin block as well. The isolation from one target area showed LOH of 17p and ROH in another region, with both fragments exhibiting *TP53 *mutations. Sequencing analysis also revealed differences in *TP53 *status. One tumour compartment (one paraffin block fragment) suggested a heterozygous mutation, whereas another exhibited a hemizygous mutation. This analysis demonstrated that the glioblastoma specimen consisted of components presenting 17p ROH and LOH, specifically, the retention and the loss of the wild-type *TP53 *allele. We conclude that cell culture under standard conditions selected for cells harbouring the *TP53 *hemizygous mutation.

### H-318 Analysis

**Figure 3:**
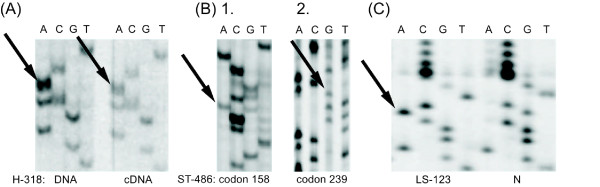
**H-318, ST-486 and LS-123 cell lines *TP53 *sequencing analysis**. **(A) **H-318 mouse adenocarcinoma cell line *TP53 *sequencing. The mutated nucleotide (*TP53 *exon 5, codon 172, CGC > CAC, Arg > His) is marked with an arrow. Only the mutated DNA is detected, suggesting a hemi/homozygous mutation. **(B) **ST-486 cell line sequencing. 1. DNA analysis showed a heterozygous mutation in codon 158 (*TP53 *exon 5, CGC > CAC, Arg > His), both mutated and wild-type nucleotides are detected. 2. Exon 7 sequencing showed the second - heterozygous mutation (*TP53*, codon 239, AAC > GAC, Asn > Asp), both nucleotides are visible. **(C) **LS-123 cell line DNA sequencing, the mutated nucleotide (*TP53 *exon 5, codon 175, CGC > CAC, Arg > His) is marked with an arrow. The analysis shows undetectable wild type DNA, suggesting homo/hemizygous mutation. Mutated nucleotides are marked with arrows. N - normal, control template.

The H-318 (adenocarcinoma) cell line was provided by Professor Guillermina Lozano, Department of Cancer Genetics, The University of Texas M. D. Anderson Cancer Center, Houston. This cell line was derived from a cancer specimen described as having a heterozygous mutation at codon 173 (equivalent of human *TP53 *codon 175) [21]. However, our DNA analysis showed the presence of only the mutated template. Consultation with the Lozano lab confirmed that the cell line was derived from a tumour showing the mutation of one allele and the retention of the second, wild-type allele (Figure 3A).

### MOLT-13 Analysis

**Figure 4:**
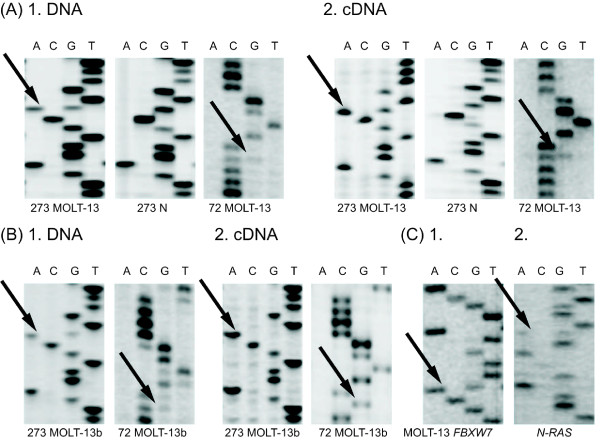
**MOLT-13 and MOLT-13 boost DNA and cDNA sequencing analysis**. **(A) **MOLT-13 *TP53*-sequencing. 1. DNA analysis shows a heterozygous mutation in codon 273 (*TP53*, exon 8, CGT > CAT, Arg > His) and a heterozygous polymorphism in codon 72 (*TP53*, exon 4, CGC > CCC, Arg > Pro), identical ratio of wild type to mutated template was detected. 2. cDNA sequencing confirms DNA results. **(B) **MOLT-13 boost *TP53*-sequencing. 1. DNA analysis shows a heterozygous mutation in codon 273 and, surprisingly, a deletion of one nucleotide - a nonsense mutation - in exon 4 (both wild type and mutated template are visible). 2. cDNA analysis showed only mutated nucleotide in codon 273 (representing hemizygous mutation) and only one allele expression in exon 4. These results suggest a missense mutation of one allele and a nonsense mutation of the other causing the nonsense mRNA decay. **(C) ***FBXW7 *and *N-RAS *DNA sequencing. In order to confirm MOLT-13 cell line identity, mutations of *FBXW7 *and *N-RAS *gene were verified. 1. *FBXW7 *analysis shows a heterozygous mutation in codon 465 (*FBXW7*, CGT > CAT, Arg > His). 2. *N-RAS *sequencing shows a heterozygous mutation in codon 12 (*N-RAS*, GGT > GAT, Gly > Asp). Mutations are marked with arrows. N - normal, control template.

The MOLT-13 cell line (T-cell leukaemia) was provided by DSMZ. Originally, the cells contained a heterozygous mutation, R273H, in *TP53 *(Figure 4A). An equivalent ratio of wild-type to mutated template was detected using DNA and cDNA analysis. After 2 months of cell culture, the wild-type cDNA was absent in the analysed sample. However, the genomic DNA still exhibited equal amounts of mutated and wild-type template at codon 273 (Figure 4B). This observation prompted us to once again perform full-gene DNA sequencing, which revealed a nonsense mutation in exon 4 (these cells were labelled MOLT-13 boost). Only one interpretation of these results was reasonable: a missense mutation of one allele and a nonsense mutation of the second allele caused nonsense-mediated mRNA decay, which was confirmed by polymorphism analysis of allele 72. Both DNA and cDNA showed traces of the original heterozygous codon 72, whereas analysis of further passages (MOLT-13 boost) uncovered a heterozygous status at the DNA level and expression of only one allele at the cDNA level (Figure 4). We considered that the second mutation could have been generated in our laboratory, or that an extremely small subpopulation of cells with the two mutations was selected. To this end, MOLT-13 cells were thawed at the earliest possible passage and again cultured for several months. After 4 weeks, we again observed the same second mutation. The population with two mutations required eight weeks to become dominant. This observation provided convincing evidence that the population with both missense and nonsense mutations was selected from cells provided by DSMZ. The identity of this cell line was confirmed by *FBXW7, PTEN, N-RAS *and STR analysis (Figure 4C).

### PF-382 Analysis

**Figure 5:**
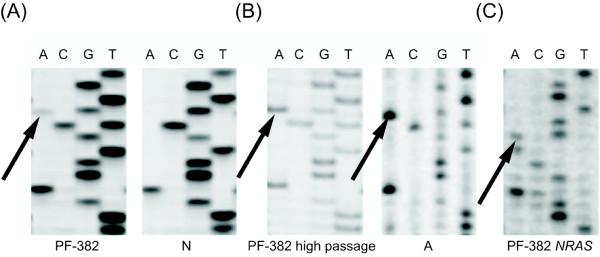
**PF-382 cell line DNA sequencing analysis**. **(A) ***TP53 *gene sequencing at passage 1. Two mutations in codon 273 are visible (nucleotide 817C > T as originally described, and surprisingly, 818 G > A). The first mutation represents heterozygous status, equal amounts of mutated and wild type allele are detected. The sequencing product shows that the wild-type nucleotide is visible as a strong band and mutated nucleotide is visible as a very weak band in the second mutation. **(B) **Cloning of PF-382 primary culture and *TP53 *sequencing after the following months of cell culturing shows that intensity of mutated band has changed. **(C) ***N-RAS *DNA sequencing. In order to confirm PF-382 cell line identity, mutation of *N-RAS *gene was verified. *N-RAS *sequencing shows a heterozygous mutation in codon 12 (*N-RAS*, GGT > AGT, Gly > Ser). Mutations are marked with arrows. N - normal, control template; A - colony.

The PF-382 cell line, derived from a T-cell leukaemia, was described as harbouring a single heterozygous mutation, R273C. DNA analysis showed two mutations at codon 273 of the *TP53 *gene (C817T and G818A). The sequencing product showed that the second mutation (818G > A) represented less than 30% of the observed template after the first passage (Figure 5A). To this end, cloning of PF-382, as well as fluorescence-activated cell sorting (FACS), was performed. Both techniques revealed that the second mutation, together with the original change, was observed only in a subpopulation of cells. SSCP analysis showed that both mutations (R273C and R273H) were localized on different alleles. Over the following months, culturing of cells produced a slow expansion of the population with both mutations (Figure 5B). The provider of the PF-382 cells did not offer information that would allow us to define the location of the second mutation. The identity of this cell line was confirmed by *FBXW7, PTEN, N-RAS *and STR analysis (Figure 5C).

### LS-123 and ST-486 Analysis

ST-486 cells were derived from a specimen of Burkitt's lymphoma, and LS-123 (large intestine adenocarcinoma) cells were originally described as carrying a heterozygous mutation in *TP53 *[18]. However, later analysis indicated the presence of two mutations in ST-486 cells. Our analysis confirmed this discovery (Figure 3B). Apparently, both mutations were present from the beginning of the ST-486 culture; however, the mutation in exon 7 was omitted, despite being present during the initial analysis.

Line LS-123 is still described as exhibiting a heterozygous mutation of *TP53 *in the Sanger database. Nonetheless, our analysis clearly shows that the wild-type allele is lacking in these cells (Figure 3C).

## Discussion

To properly judge the importance of heterozygous mutations in *TP53*, an appropriate model for its analysis is required. Heterozygosity of *TP53 *mutations is the basis of the dominant-negative effect. For the most part, the analyses of this phenomenon have been performed in artificial systems and using animal models. However, co-transfection of wild-type and mutated cDNA is artificial and does not allow for the analysis of all aspects of gene function, and animal models do not represent exact human counterparts [7]. To this end, the analysis of human cancer cell lines with single heterozygous *TP53 *mutations could be an alternative. However, this approach features some caveats as well. Despite the fact that a percentage of surgical and biopsy specimens that are described as harbouring a single heterozygous mutation is very high (35%), the proportion of cultured cancer cell lines supposedly carrying such a mutation is lower (10%) (Figure 1A, 1B). Moreover, based on our analysis, even this number appears to be overestimated. These differences should be investigated in order to estimate the importance of the dominant-negative effect of *TP53 *mutations. A higher percentage of single heterozygous mutations indicates a greater importance of dominant-negative effect, as has been shown for genes such as *IDH1 *or *EGFR *[13,14].

First, genetic heterogeneity can sometimes be mistaken for the heterozygous status of the *TP53 *gene. In cancer specimens exhibiting a *TP53 *mutation, cells without a mutation are present as well. *TP53 *is mutated at the end of the mutation pathway, and many cancer cells without a mutation in this gene can be observed within the specimen [22-25].

Another explanation for the discussed discrepancies may be the more thorough genetic analysis of *TP53 *gene sequences in cell lines compared to tumour samples, which implies that cell line studies are more reliable in this respect. For the most part, tumour samples (especially where paraffin blocks are concerned) do not provide a sufficient amount of high-quality DNA to repeatedly analyse all *TP53 *gene elements. The percentage of single heterozygous mutations is lower than suggested by *in vivo *analysis, and thus, the importance of the DNE of *TP53 *mutations is undermined. Database analysis supports such a possibility. Interestingly, cell lines exhibit a lower proportion of wild-type *TP53 *retention, not merely because of the more frequent 17p LOH, but also due to the more frequent second heterozygous *TP53 *mutation (Figure 1C). The percentage of samples with at least one mutation outside exons 5-8 is higher in cancer cell lines with at least two mutations than in cancer surgical samples with two mutations (42% in cell lines and 31% in tumour samples) (Figure 1D). Although the difference is not statistically significant, the investigation of this discrepancy leads to the suspicion that some of the mutations outside region 5-8 were not detected due to negligence during the analysis of surgical and biopsy samples. It has been confirmed in many cell line analyses that the first examination is not sufficiently thorough, whereas surgery/biopsy sample analyses are rarely repeated [26-28]. The TE-3 cell line is a good example of this issue. In the first paper, the lack of a mutation was suggested [26]; however, an additional analysis revealed a homozygous splice-site mutation in intron 4 of *TP53 *[27]. Importantly, TE-3 cells were initially shown as lacking the TP53 protein [26]. Apparently, an undetected homozygous nonsense mutation was the reason for the absence of TP53 protein. This problem can also be demonstrated using the ST-486 cell line. Originally described as exhibiting one heterozygous mutation, ST-486 has currently been redefined as harbouring two mutations [28]. Our analysis confirmed the presence of the two mutations in ST-486 cells (Figure 3B). The data presented here suggest the overestimation of the proportion of heterozygous *TP53 *mutations, both *in vivo *and *in vitro*. Notwithstanding, this information cannot fully account for the discussed (*in vivo - in vitro*) *TP53 *discrepancies, which are crucial for estimating the dominant-negative effect of *TP53 *mutations. Intriguingly, the list of cell lines showing a single heterozygous mutation of *TP53 *is diminishing. The process of cell line reclassification is currently very vigorous. The Sanger Database features many cell lines that have changed status within the last 2 years, from having a single heterozygous mutation to exhibiting more than one mutation (*vide *cell lines: CMK, Cha-Go-K-1 and NCI-H661). It is highly unlikely that each pair of double mutations affected the same allele. Moreover, some cell lines are reported differently between databases: MOLT-16, KM-12, SK-LMS-1, J-82, Daudi and Raji. Notably, the LS-123 cell line, which is still catalogued as heterozygous, was provided to us with a hemizygous *TP53 *mutation (Figure 3C). The reasons for these on-going changes in the databases are not clear because we are unable to determine whether the late discovery of the second mutation is the result of its generation *in vitro*, selection of an originally small subpopulation of cells with this mutation *in vivo*, experimental errors or technical inaccuracies. The difficulty in discriminating between the first two potential explanations will be the subject of the following paragraphs.

**Figure 6:**
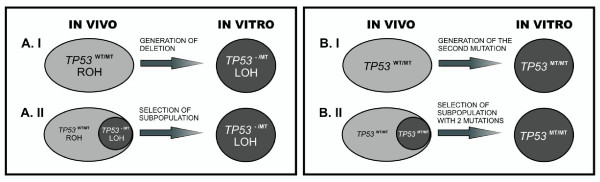
**Two proposed hypotheses (generation and selection)**. **(A) ***In vitro *generation of 17p LOH or *in vitro *occurring selection of small subpopulation of cells without wild type *TP53 *allele. **(B) ***In vitro *mutation of allele showing *in vivo *normal sequence or *in vitro *occurring selection of small subpopulation of cells with 2 mutated *TP53 *alleles.

The *in vitro *selection of cells exhibiting a hemizygous mutation "hidden" within cells with a heterozygous mutation and the generation of 17p LOH or of a new point mutation in the other allele (initially/*in vivo *wild-type allele) may be vital in elucidating the lack of a single *TP53 *heterozygous mutation in cell lines (Figure 6). Our experimental analyses addressed both issues.

Data published by Lozano *et al. *and their analysis of the H-318 cell line, which showed ROH of 17p, support the latter scenario [21]. Nevertheless, our investigation demonstrated the lack of the wild-type DNA template (Figure 3A). These results suggest the elimination of the wild-type allele during culturing. The conclusions of Lozano *et al. *are modestly supported by the analysis performed by Boyle *et al*. The loss of the wild-type allele i*n vitro *was well defined during the examination of fibroblasts derived from patients with Li-Fraumeni syndrome [29]. These cells originally showed only a heterozygous mutation, whereas after 15-20 passages, colonies with 17p LOH were detected. Subsequently, the cells with 17p LOH became dominant [29]. The analysis of Li-Fraumeni patients' fibroblasts supports Lozano's conclusion; however, it is not certain whether the H-318 cell line derives from the selection for cells with a hemizygous mutation or the generation of 17p LOH *in vitro*.

The phenomenon of the selection for cells with a hemizygous mutation was exemplified by the derivation of the G-16 cell line by our laboratory. This analysis showed the presence of at least two neoplastic compartments, one with the heterozygous and the other with the hemizygous mutation of *TP53*. This strongly suggests that selection for cells with the hemizygous mutation, as opposed to its *de novo *generation, was responsible for the discrepancies between the surgical sample and the established cell line. The aforementioned selection of cells without the wild-type *TP53 *allele indicates that the dominant-negative effect of *TP53 *mutations is less important than is generally suggested. Clearly, a heterozygous mutation is not as influential as a hemizygous, homozygous or double heterozygous mutation. In some cases, we were not able to precisely define whether the mutation was generated *in vitro *or selection had occurred. Nevertheless, the cell lines described in the databases as carrying a single heterozygous mutation appeared very unstable in this state or were potentially misclassified. This may be exemplified by the analysis of PF-382 cells. Our study of this cell line revealed two mutations in codon 273 (Figure 5), although the databases indicate only a single mutation. One of these mutations was originally observed only in a subpopulation of cells. We could not exclude that a subpopulation of cells exhibiting two mutations was already present *in vivo *and was later ignored during the original DSMZ investigation. Alternatively, the second mutation may have been generated during the production of cell culture stocks. SSCP analysis and cell cloning for PF-382 cells support the conclusion that both mutations in codon 273 affected different alleles.

The analysis of another cell line demonstrated similar results. The MOLT-13 cell line was provided to our laboratory by DSMZ, and only one heterozygous mutation in codon 273 of the *TP53 *gene was detectable in the early passages. After 2 months of culturing under standard conditions, the second mutation was detected during a routine screening (Figure 4B), which prompted us to restart the culture of the original MOLT-13 cells. Culturing these cells for only four weeks produced cells with both mutations, which established that the presence of cells with the two mutations was the result of selection in our laboratory, rather than *de novo *generation. The data provided by DSMZ did not allow us to exclude that the second mutation was not present in the original patient sample and was generated during the production of vendor stocks. In any case, if generation or selection occurs *in vitro*, the importance of the dominant-negative effect is undermined by the instability of single heterozygous mutations, as well as the enhanced *in vitro *survival of cells exhibiting a lack of wild-type *TP53*.

The data presented above show that although surgical specimens are analysed less precisely than cell lines, single heterozygous mutations are still observed more frequently *in vivo *than *in vitro*. The G16, MOLT13-boost and PF-382 cell lines consist of cells that were selected from subpopulations arising *in vivo *(in the case of the MOLT13-boost minor subpopulation), whereas H-318 cells probably acquired the 17p LOH *in vitro *(adaptation to *in vitro *conditions and further stages of tumorigenesis). Thus, it may be presumed that it is possible to detect many biological differences between cells observed *in vivo *and *in vitro *in terms of *TP53 *status. For example, artificial selection forces acting *in vitro *may change the *TP53 *status. Alternatively, rapid selection and generation of subpopulations of the more advanced neoplastic cells may be observed under such conditions. The latter interpretation would favour *in vitro *conditions as selecting the most effective impairment of *TP53*, i.e., the selection of cells at more advanced stages of carcinogenesis.

Nonetheless, an effective DNE would not be easily replaced by elimination or impairment of the second allele under artificial or *in vivo *selection pressure. Moreover, it is very unlikely that a mechanism that is sufficiently effective *in vivo *would become utterly suppressed *in vitro*. In general, *in vitro *selection and generation of cells presenting hemi/homozygous mutations undermines the importance of dominant-negative inactivation of *TP53*. Still, we cannot exclude that *in vitro *cell culture does not optimally recapitulate all aspects of tumorigenesis involving *TP53*.

We are aware that DNE has been presented many times for some of the *TP53 *mutations. Our analysis suggests that DNE *TP53 *mutations, which are albeit limited in general, differ with respect to specific mutations. First, the mutation R175H seems to exhibit a weaker DNE than mutations in codon 248. Furthermore, our results are very intriguing for DNE mutations in codon 273 (R273L, R273P, R273H) in comparison to R273C, a mutation exhibiting a lack of DNE, as demonstrated by Dearth *et al*. In part, it could be expected that the percentage of single heterozygous mutations correlates with the magnitude of DNE and the importance of specific dominant-negative mutations defined by Dearth *et al*. Namely, the percentage of mutations in codon 273 defined by Dearth *et al. *as exhibiting a DNE was higher *in vivo *than this number for the R273C mutation (42% vs. 23%). On the other hand, the percentage of single heterozygous R273C mutations was 4 times higher *in vitro *than other mutations in this codon (20% vs. 5%), undermining the importance of *TP53 *mutations DNE *in vitro*. Nevertheless, segregating *TP53 *mutations into hot spots changes the overall results very little. None of the hot spots exhibit a percentage of single heterozygous mutations higher than 50% (Figure 1F), bearing in mind that these values are overestimated according to our analyses. Still, the data do not imply a complete lack of DNE. It may be inferred that DNE is a mechanism operating solely *in vivo*, which, however, remains in contradiction with the DNE model. Moreover, other tumour suppressor genes, such as *APC *and *Rb*, also show similar differences between the frequency of single heterozygous mutations *in vivo *and *in vitro *[17,18]. Obviously, *APC *and *Rb *exhibit nonsense mutations, and DNE is not attributed to these genes, whereas epigenetic silencing is [30-33]. However, considering the latter observation, another analysis of the database is very appealing. The retention of the wild-type allele in cancer cell lines with a missense *TP53 *mutation is almost the same as the frequency amongst cancer cell lines with a nonsense mutation (Figure 1E). This indicates that a missense mutation does not predispose cells to retain the wild-type *TP53 *allele any more than does a nonsense mutation, whereas a dominant-negative effect is expected to be the attribute of missense mutations only. In our opinion, this suggests that the dominant-negative effect can be confused with gain of function and even mistaken for an unknown mechanism of wild-type *TP53 *inhibition if a single heterozygous mutation occurs. Our group has already demonstrated the predominance of mutated mRNA over wild-type mRNA in glioblastoma specimens showing putative single heterozygous mutation of *TP53 *[20].

## Conclusion

Considering all the data presented here, the DNE of *TP53 *mutations seems to be less important than the majority of reports that describe this phenomenon suggest. Nevertheless, this effect should not be ignored, and we do not exclude that it plays an important role at the earliest stages of tumorigenesis.

In conclusion, we summarize the following facts discussed in our article undermining the influence of the dominant-negative effect of *TP53 *missense mutations:

- the percentage of *TP53 *single heterozygous mutations described in databases is low (35% *in vivo *and 10% *in vitro*),

- the percentage of single heterozygous mutations in cancer specimens (*in vivo) *is overestimated in databases,

- the percentage of single heterozygous mutations in cancer cell lines (*in vitro) *is overestimated in databases,

- cancer cells exhibiting a lack of wild-type *TP53 *are generated and/or selected for *in vitro*,

- the percentage of single heterozygous mutations of *TP53 *is higher in cancer specimens than in cancer cell lines,

- mutations considered to show DNE, R175H and R273X (excluding R273C), represent about 5% of single heterozygous mutations *in vitro*, according to the IARC database,

- the frequency of retention of the wild-type allele in cancer cell lines and tumour samples with a missense mutation is almost the same as the frequency amongst cancer cell lines and tumour samples with a nonsense mutation, according to databases.

## Competing interests

The authors declare that they have no competing interests.

## Authors' contributions

ESF conceived study design, performed database and genetic analyses, wrote the manuscript, contributed to data interpretation and cell culturing. MS performed genetic analyses, sequenced *TP53 *and contributed to data interpretation. SP participated in the conception and design of the study, genetic analyses and data interpretation. MiB participated in the study design, genetic analyses, provided help in manuscript preparation and data interpretation, performed the database and statistical analysis. KHB was responsible for cell culturing. MaB carried out genetic analyses and database analysis. IZ participated in the acquisition of funding, contributed to genetic analyses and data interpretation. DJK and RK provided histopathology results and participated in data interpretation. PPL participated in the manuscript preparation, revised the manuscript critically. PR supervised the project, conceived study design and provided help in manuscript preparation, participated in the acquisition of funding, contributed to data interpretation and cell culture. All authors have given final approval of the version to be published.

## Pre-publication history

The pre-publication history for this paper can be accessed here:

http://www.biomedcentral.com/1471-2407/11/243/prepub
